# Reasons of not having breast reconstruction: a historical cohort of 1937 breast cancer patients undergoing mastectomy

**DOI:** 10.1186/2193-1801-2-325

**Published:** 2013-07-18

**Authors:** Delphine Héquet, Kevin Zarca, Sylvie Dolbeault, Benoît Couturaud, Charlotte Ngô, Virgine Fourchotte, Anne De La Rochefordière, Jean-Guillaume Féron, Alfred Fitoussi, Catherine Bélichard, Fabien Reyal, Fatima Laki, David Hajage, Brigitte Sigal, Bernard Asselain, Séverine Alran, Institut Curie Breast Cancer Group, Roman Rouzier

**Affiliations:** Department of Surgery, Paris, France; Department of Biostatistics, Paris, France; Department of Psycho-oncology, Paris, France; Department of Radiotherapy and Pathology, Paris, France; Institut Curie, 26 rue d’Ulm, Paris, 75005 France

**Keywords:** Breast cancer, Mastectomy, Breast reconstruction, Personal choice, Medical information

## Abstract

**Background:**

The aims of the study were to investigate the factors associated with not having breast reconstruction following mastectomy and to assess patient satisfaction with information on reconstruction.

**Patients and methods:**

We analysed a historical cohort of 1937 consecutive patients who underwent mastectomy at Institut Curie between January 2004 and February 2007. Their sociodemographic and clinicobiological characteristics were recorded in a prospective database. A questionnaire was sent to 10% of nonreconstructed patients.

**Results:**

The proportion of patients with invasive cancer was 82.7%. The rate of nonreconstruction in patients with in situ and invasive cancer was 34.6% and 74.9%, respectively. On multivariate analysis, only employment outside the home was associated with reconstruction in patients with in situ cancer (p < 0.001). In patients with invasive cancer, employment status (p < 0.001) and smoking (p = 0.045) were associated with reconstruction, while age > 50, ASA score >1, radiotherapy (p < 0.0001) and metastatic status (p = 0.018) were associated with nonreconstruction. For 80% of questionnaire responders, nonreconstruction was a personal choice, mainly for the following reasons: refusal of further surgery, acceptance of body asymmetry, risk of complications and advanced age. Information on reconstruction was entirely unsatisfactory or inadequate for 62% of patients.

**Conclusion:**

Better understanding the factors that influence decision of nonreconstruction can help us adapt the information to serve the patient’s personal needs.

## Introduction

About 52,500 women are diagnosed with breast cancer in France every year (La situation du cancer en France 2010). Breast-conserving therapy is standard procedure for selected patients (Fisher et al. 2002; Veronesi et al. 1995), however, between 2005 and 2009, 26% of breast cancer patients in France underwent mastectomy (La situation du cancer en France 2010). Only 22.9% of patients who undergo mastectomy in France proceed with reconstruction (PMSI national 2011).

Mastectomy and reconstruction rates vary widely from between countries, regionally within countries and over time, making it difficult to produce meaningful comparisons. Nevertheless, what emerges across all studies is that the majority patients do not have reconstruction following mastectomy (Table 1) (Kruper et al. 2011; Jeevan et al. 2010; Hvilsom et al. 2011; Morrow et al. 2001; Reaby 1998; Baxter et al. 2005; Yu et al. 2007; Meyer-Marcotty et al. 2007; Fallbjork et al. 2010; Al-Allak et al. 2010; Al-Ghazal et al. 2000; Harcourt et al. 2003; Christian et al. 2006; Joslyn 2005; Rowland et al. 2000). Numerous studies have looked at the techniques and timing of breast reconstruction, patient satisfaction and quality-of-life after reconstruction, as well as the relative mastectomy/reconstruction rates, and the factors affecting these rates. These studies tend to focus on patients who undergo reconstruction. A few studies have given equal importance to patients who do not have reconstruction (Morrow et al. 2001; Reaby 1998; Fallbjork et al. 2010; Harcourt et al. 2003; Alderman et al. 2003).

**Table 1:** **Reconstruction rates in various countries**

Author	Country	Type of study sample size	Mastectomy rate	Reconstruction rate
Reaby et al. (1998)	Australia	Questionnaire N = 95	–	10%
Baxter et al. (2005)	Canada	Retrospective N = 27 718	42.9%	7.9%
Yu et al.(2007)	China	Retrospective N = 5887	93.7%	5%
Hvilsom et al. (2011)	Denmark	National registry N = 13 379	37%	25%
Meyer-Marcotty et al. (2007)	Germany	Retrospective N = 4335	34%	13%
Fallbjork et al. (2010)	Sweden	Questionnaire N = 149	40%	25%
Al-Allak et al.(2010)	United Kingdom	Retrospective N = 272	–	46%
Al-Ghazal et al. (2000)	United Kingdom	Retrospective N = 577	35%	21%
Harcourt et al. (2003)	United Kingdom	Prospective N = 103	–	46%
Jeevan et al. (2010)	United Kingdom	Registry N = 44 837	40%	16%
Christian et al. (2006)	United States	National registry N = 2174	42%	42%
Joslyn (2005)	United States	National registry N = 27 703	–	17%
Kruper et al. (2011)	United States	State registry N = 11 019	42%	25-29%
Morrow et al. (2001)	United States	National registry N = 68 348	–	8.3%
Rowland et al. (2000)	United States	Retrospective N = 1957	43%	17%
**Our study Héquet et al.**	**France**	**Retrospective N = 1937 Questionnaire N = 132**	**26%**	**32.1%**

To our knowledge, this was the first recent study that proposed to focus specifically on patients who did not have surgical reconstruction. The aim was to gain a better understanding of the factors, both medical and personal, that led to the decision and to consider the implications in terms of patient management and counseling. To that end, we assessed the sociodemographic and clinicobiological factors associated with surgical reconstruction in patients who did not have reconstruction and in patients who did, whether immediate or delayed. In addition, we investigated the reasons why patients did not have surgical reconstruction, and evaluated the information provided to these patients.

## Patients and methods

### Study population

Consecutive patients treated by mastectomy for in situ or invasive breast cancer between January 2004 and December 2007 in Institut Curie (Paris, France), were eligible for inclusion. Exclusion criteria were history of contralateral mastectomy, bilateral mastectomy, mastectomy for benign disease, prophylactic mastectomy, and men. The study was approved by the Breast Cancer Study Group of Institut Curie, and the questionnaire was approved by the Ethics Committee of Institut Curie.

### Data collection

Data in the medical charts of a historical cohort of 1937 patients were prospectively recorded for analysis. The following factors were studied: age at mastectomy, marital status, professional status, weight (in kg), height (in cm), breast size, American Society of Anesthesiologists (ASA) score, histological grade of cancer (in situ carcinoma, invasive carcinoma grade I, II, III of the Scarff-Bloom-Richardson classification), tumor size (in cm), estrogen-receptor status, progesterone-receptor status, HER2 status, axillary lymph node status, indication of mastectomy (for clinicobiological factors of cancer, after failure of conservative treatment, or for recurrence), and adjuvant therapy (hormone therapy, chemotherapy, radiotherapy).

### Questionnaire

A questionnaire was sent to a 10% sample of patients who did not have reconstruction (*n* = 132), including every tenth patient in the retrospective database. The questionnaire was prepared in collaboration with a panel of 8 patients who had undergone mastectomy only and a panel of medical and paramedical professionals working in the field of breast cancer. The questionnaire was sent out in June 2011. Responders remained anonymous. All questions were multiple choice, and patients could provide more than one response. At the end of the questionnaire, free-text comments could be added. The responses were analyzed descriptively, and free-text comments were collected.

### Analysis

The sociodemographic and clinicobiological factors associated with reconstruction were assessed in two groups: patients who did not have reconstruction and patients who had immediate or delayed reconstruction. Univariate analysis was performed using Chi-square test for categorical variables and Student’s *t*-test for continuous variables. Multivariate analysis was performed using logistic regression models on two groups: patients with in situ cancer and patients with invasive cancer. Variables associated with reconstruction (*P* < 0.10) in the univariate analysis were introduced into the multivariate model, as more traditional approaches, such as considering all variables with *P* < 0.05 in the univariate analysis, can fail to identify variables known to be important. A *P*-value < 0.05 was considered statistically significant.

## Results

### Description of the cohort

Among the 1937 patients included, 1315 (67.9%) had no surgical reconstruction and 622 (32.1%) patients had surgical reconstruction, which was immediate in 363 patients (58.5%) and delayed in 259 patients (41.5%). Among patients who ultimately had delayed reconstruction, the median time to reconstruction was 19 months (range: 3–77 months).

Patient characteristics are summarized in Table 2.

**Table 2:** **Characteristics of the population**

	All patients (N = 1937)
Age (years) - median (range)	56 (23–97)
Marital status - N (%)	
*Not partnered*	411 (21.3)
*Partnered*	651 (33.6)
*Unknown*	875 (45.1)
Employment status - N (%)	
*Not working outside home*	609 (31.4)
*Working outside home*	900 (46.5)
*Unknown*	428 (22.1)
BMI^a^ (kg/m^2^) - N (%)	
*<20*	292 (15.1)
*20-25*	925 (47.8)
*26-30*	450 (23.2)
*>30 Unknown*	221 (11.4) 49 (2.5)
Breast size (cm) - median (range)	90 (65–130)
Cup size - N (%)	
*A*	108 (5.6)
*B*	521 (26.9)
*C*	299 (15.4)
*≥D*	183 (9.5)
*Unknown*	826 (42.6)
Tobacco users - N (%)	296 (15.3)
ASA^b^ score - N (%)	
*ASA 1*	1051 (54.2)
*ASA 2*	824 (42.6)
*ASA >2*	62 (3.2)
Histologic stage - N (%)	
In situ	335 (17.3)
*SBR*^c^*I*	217 (11.2)
*SBR II*	716 (37)
*SBR III*	614 (31.7)
*Invasive SBR unknown*	54 (2.8)
Tumor size (cm) - N (%)	
*<2*	603 (31.2)
*2-5*	919 (47.5)
*>5*	316 (16.3)
*Unknown*	98 (5)
Estrogen receptors positive - N (%)	1251 (64.6)
Progesterone receptors positive - N (%)	444 (22.9)
Overexpression HER2 - N (%)	228 (11.8)
Axillary lymph nodes positive - N (%)	726 (37.5)
Inflammatory cancer - N (%)	92 (4.8)
Metastatic cancer - N (%)	83 (4.3)
Indication of mastectomy - N (%)	
*Clinicobiological characteristics of cancer*	1061 (54.8)
*Failure conservative surgery*	357 (18.4)
*Local recurrence*	519 (26.8)
Chemotherapy - N (%)	
*Adjuvant*	715 (36.9)
*Neoadjuvant*	284 (14.7)
Radiotherapy - N (%)	971 (50.2)
Hormone therapy - N (%)	1175 (60.7)

^a^*BMI* body mass index.

^b^*ASA* American Society of Anesthesiologists.

^c^*SBR* Scarff-Bloom-Richardson grade.

### Factors associated with not having surgical breast reconstruction

#### In situ cancer

Mastectomy was indicated for in situ cancer in 335 patients (17.3%). The nonreconstruction rate was 34.6%. The reconstruction rate was 65.4% immediate in 82.6% of cases and delayed in only 17.4% of cases. Univariate analysis showed a statistical difference between reconstructed and nonreconstructed patients for several factors (Table 3). Reconstructed patients were 7 years younger than nonreconstructed patients (*P* < 0.001). Not working outside the home was more frequent in nonreconstructed patients (48.9% versus 21.3%, *P* < 0.001). Twenty-eight percent of reconstructed patients had an ASA score above 1, while 44.8% of nonreconstructed patients had a score above 1 (*P* = 0.004).

**Table 3:** **Univariate analysis in 335 patients with in situ cancer**

	Nonreconstructed N = 116	Reconstructed N = 219	P-value
Age (years) - median (range)	57 (23–86)	50 (26–75)	**< 0.001**
Marital status - N (%)			0.134
*Not partnered*	28 (42.4)	40 (30.5)	
*Partnered*	38 (57.6)	91 (69.5)	
Employment status - N (%)			**< 0.001**
*Not working outside home*	46 (48.9)	39 (21.3)	
*Working outside home*	48 (51.1)	144 (78.7)	
BMI^a^ (kg/m^2^) - N (%)			0.391
*<20*	23 (20.2)	42 (19.3)	
*20-25*	59 (51.8)	123 (56.4)	
*26-30*	20 (17.5)	41 (18.8)	
*>30*	12 (10.5)	12 (5.5)	
Tobacco users - N (%)	15 (12.9)	38 (17.4)	0.369
ASA^b^ score - N (%)			**0.004**
*ASA 1*	64 (55.2)	157 (71.7)	
*ASA 2*	48 (41.4)	60 (27.4)	
*ASA >2*	4 (3.4)	2 (0.9)	
Tumor size (cm) - N (%)			0.123
*<2*	29 (27.1)	41 (23.7)	
*2-5*	56 (52.3)	77 (44.5)	
*>5*	22 (20.6)	55 (31.8)	
Indication of mastectomy - N (%)			0.612
*Clinicobiological characteristics of cancer*	57 (49.1)	96 (43.8)	
*Failure conservative surgery*	39 (33.6)	78 (35.6)	
*Local recurrence*	20 (17.2)	45 (20.5)	

^a^*BMI* body mass index.

^b^*ASA* American Society of Anesthesiologists.

In the multivariate analysis, only employment status was statistically different between the 2 groups, with 4 times more patients in the reconstructed group working outside the home (OR: 4.05; CI: 2.05-8.00; *P* < 0.001). No statistically significant difference was found for the other criteria (Table 4).

**Table 4:** **Multivariate analysis for breast reconstruction**

	Patients with in situ cancer N = 335			Patients with invasive cancer N = 1602		
	OR^a^	CI^b^	P-value	OR	CI	P-value
Age (years)			0.091			**< 0.001**
*<35*	1.00			1.00		
*35-50*	0.92	0.23-3.72		0.45	0.24-0.86	
*>50*	0.45	0.11-1.84		0.22	0.11-0.44	
Employment status			**< 0.001**			**< 0.001**
*Not working outside home*	1.00			1.00		
*Working outside home*	4.05	2.05-8.00		2.07	1.37-3.13	
BMI^c^ (kg/m^2^)			0.420			0.078
*<20*	1.00			1.00		
*20-25*	1.79	0.80-4.00		1.30	0.83-2.02	
*26-30*	2.21	0.81-6.03		0.80	0.46-1.38	
*>30*	1.91	0.53-6.84		1.50	0.80-2.82	
Tobacco users						**0.045**
No				1.00		
Yes				1.52	1.01-2.28	
ASA^d^ score			0.155			**< 0.001**
*ASA 1*	1.00			1.00		
*ASA 2*	0.63	0.33-1.19		0.51	0.36-0.73	
*ASA >2*				0.00		
Tumor size (cm)			0.232			0.655
*<2*	1.00			1.00		
*2-5*	0.58	0.27-1.24		1.09	0.75-1.58	
*>5*	0.94	0.39-2.25		1.28	0.76-2.14	
Metastatic cancer						**0.018**
No				1.00		
Yes				0.34	0.13-0.91	
Indication of mastectomy						**0.024**
*Clinicobiological characteristics of cancer*				1.00		
*Failure conservative surgery*				1.27	0.84-1.91	
*Local recurrence*				2.09	1.23-3.55	
Chemotherapy						0.748
No				1.00		
*Adjuvant*				0.94	0.60-1.47	
*Neoadjuvant*				0.81	0.46-1.43	
Radiotherapy						**< 0.001**
No				1.00		
Yes				0.57	0.38-0.86	

^a^*OR* odds ratio.

^b^*CI* confidence interval.

^c^*BMI* body mass index.

^d^*ASA* American Society of Anesthesiologists.

#### Invasive cancer

Mastectomy was indicated for invasive cancer in 1602 patients (82.7%). The nonreconstruction rate was 74.9%. The reconstruction rate was 25.1% immediate in 45% of cases and delayed in 55% of cases.

Univariate analysis showed that reconstructed patients were 9 years younger than nonreconstructed patients (Table 5). Forty-nine percent of nonreconstructed patients were not working outside the home, compared to only 22% of reconstructed patients (*P* < 0.001). High Body Mass Index (>25 kg/m^2^) was more frequent in nonreconstructed patients (41.1% versus 27.7%, *P* < 0.001). Smoking was more frequent in reconstructed patients (20.9% versus 13.3%, *P* < 0.001). Positive axillary lymph-node or metastatic status was more frequent in nonreconstructed patients (48.3% versus 36.5%, *P* < 0.001 and 6.4% versus 2.2%, *P* = 0.002 respectively). Radiotherapy was more frequent in nonreconstructed patients (60% versus 52.7%, *P* = 0.012).

**Table 5:** **Univariate analysis in 1602 patients with invasive cancer**

	Nonreconstructed N = 1200	Reconstructed N = 402	P-value
Age (years) - median (range)	59.6 (28–97)	50.5 (25–76)	**< 0.001**
Marital status - N (%)			0.171
*Not partnered*	266 (41.1)	77 (35.5)	
*Partnered*	382 (59)	140 (64.5)	
Employment status - N (%)			**< 0.001**
*Not working outside home*	457 (49.4)	67 (21.9)	
*Working outside home*	469 (50.6)	239 (78.1)	
BMI^a^ (kg/m^2^) - N (%)			**< 0.001**
*<20*	158 (13.6)	69 (17.5)	
*20-25*	527 (45.3)	216 (54.8)	
*26-30*	317 (27.3)	72 (18.3)	
*>30*	160 (13.8)	37 (9.4)	
Tobacco users - N (%)	159 (13.3)	84 (20.9)	**< 0.001**
ASA^b^ score - N (%)			**< 0.001**
*ASA 1*	557 (46.5)	272 (67.7)	
*ASA 2*	588 (49)	128 (31.8)	
*ASA >2*	54 (4.5)	2 (0.05)	
Tumor size (cm) - N (%)			**0.004**
*<2*	377 (32)	156 (40.9)	
*2-5*	619 (52.6)	167 (43.8)	
*>5*	181 (15.4)	58 (15.2)	
Axillary lymph nodes positive N (%) Median (range)	579 (48.3%) 1 (0–28)	147 (36.5) 0 (0–19)	**< 0.001**
Metastatic cancer - N (%)	76 (6.4)	9 (2.2)	**0.002**
Indication of mastectomy - N (%)			**< 0.001**
*Clinicobiological characteristics of cancer*	720 (60.1)	187 (46.5)	
*Failure conservative surgery*	162 (13.5)	78 (19.4)	
*Local recurrence*	317 (26.4)	137 (34.1)	
Chemotherapy - N (%)			0.253
*Adjuvant*	501 (41.8)	187 (46.5)	
*Neoadjuvant*	208 (17.3)	64 (15.9)	
Radiotherapy - N (%)	720 (60)	212 (52.7)	**0.012**
Hormone therapy - N (%)	853 (71.1)	283 (70.4)	0.825

^a^*BMI* body mass index.

^b^*ASA* American Society of Anesthesiologists.

In the multivariate analysis, reconstruction was less frequent when the following factors were present: age over 50 (OR: 0.22; CI: 0.11-0.44; *P* < 0.001), ASA score above 1 (OR: 0.51; CI: 0.36-0.73; *P* < 0.001), metastatic status (OR: 0.34; CI: 0.13-0.91; *P* = 0.018), and radiotherapy (OR: 0.57; CI: 0.38-0.86; *P* < 0.001). The following factors were associated with reconstruction: working outside the home (OR: 2.07; CI: 1.37-3.13; *P* < 0.001), smoking (OR: 1.52; CI: 1.01-2.28; *P* = 0.045), HER2 overexpression (OR: 1.75, CI: 1.13-2.70, *P* = 0.029), and mastectomy indicated for local recurrence (OR: 2.09; CI: 1.23-3.55; *P* = 0.024). The results are presented in Table 4.

### Reasons for not having surgical reconstruction

The response rate to the questionnaire was 61.4% (81 patients).

Forty-nine patients (80%) declared that the decision not to have reconstruction was a personal choice. Eleven patients (18%) declared that reconstruction was not offered by the surgeon. Twelve patients (19.7%) declared that the decision was a personal choice and that reconstruction was not offered by the surgeon. Five patients (8.2%) declared that reconstruction was not undertaken for medical reasons.

The reasons for deciding not to have reconstruction, among patients who declared that the decision was a personal choice, are shown in Table 6.

**Table 6:** **Analysis of questionnaire data**

	N	%
Questionnaires sent out	132	100
Questionnaires returned	81	61.4
*Non-respondents (*i.e. *refused to participate, deceased or subsequently reconstructed)*	20	24.7
*Respondents*	61	75.3
Reason for nonreconstruction		
*Personal choice*	33	54.1
*Not offered by surgeons and personal choice*	12	19.7
*Not offered by surgeons*	11	18
*Medical reasons and personal choice*	4	6.6
*Medical reasons*	1	1.6
Personal choice		
*Refusal of further surgery*	36	59
*Acceptance of body asymmetry*	23	37.7
*Risk of complications*	18	29.5
*Advanced age*	14	23
*Fear of masking recurrence*	11	18
*Acceptance of body asymmetry by partner*	11	18
*Financial cost*	9	14.8
*Post-mastectomy pain*	4	6.6
Source of information on reconstruction		
*Surgeon at IC*^*a*^	21	34.4
*Plastic surgeon at IC*	8	13.1
*Physician outside IC*	7	11.5
*Physician or nurse at IC*	7	11.5
*Relative with history of reconstruction*	6	8.8
*Internet, review, magazine*	5	8.2
*Patients association*	3	4.9
*IC website or review*	3	4.9
*Plastic surgeon outside IC*	2	3.3
Satisfaction with information on reconstruction		
*Entirely unsatisfactory*	25	41
*Inadequate*	13	21.3
*Adequate*	17	27.9
*Entirely satisfactory*	6	9.8

^a^*IC* Institut Curie.

### Evaluation of information on surgical breast reconstruction

Analysis of the responses to questions concerning the source of information on reconstruction and patient satisfaction is shown in Table 6.

## Discussion

In France, the reconstruction rate is around 23% (PMSI national 2011). The rate is 32.1% at Institut Curie, a national cancer institute in Paris. Other studies have shown that reconstruction rates tend to be higher in specialized cancer-treatment centers (Kruper et al. 2011; Jeevan et al. 2010; Hvilsom et al. 2011; Morrow et al. 2001), and our study supports this finding, with a reconstruction rate well above the national average.

Previous studies have shown that younger patients are more likely to have reconstruction compared to older patients (Kruper et al. 2011; Jeevan et al. 2010; Morrow et al. 2001; Reaby 1998; Fallbjork et al. 2010; Al-Allak et al. 2010; Christian et al. 2006; Joslyn 2005; Rowland et al. 2000). In our study, age over 50 was significantly associated with nonreconstruction in patients with invasive cancer, but only approached significance in patients with in situ cancer. It has been theorized that this difference may reflect physicians’ attitudes, the fact that older women attach less importance to body image, or that they have more comorbidities. A recent review on reconstruction in older patients cites the fear of complications as a major dissuasive factor, even though studies have shown that age in itself is not a risk factor for poor surgical outcomes (Walton et al. 2011).

Pathology tumor stage has also been shown to be an important predictor of nonreconstruction (Morrow et al. 2001; Christian et al. 2006; Joslyn 2005). In our study, patients with primary invasive cancer were much less likely to undergo reconstruction than those with in situ cancer or local recurrence. The probable explanation is that, in the latter two cases, reconstruction can be performed at the same time as mastectomy, thereby limiting the number of additional surgical interventions, as illustrated by the fact that, in almost 83% of patients with in situ cancer who had reconstruction, the procedure was immediate. In addition, these patients did not receive post-operative radiotherapy. In our study, in patients with invasive cancer, radiotherapy significantly reduced the odds of having reconstruction, however confounding factors may be present, including cancer stage and type of reconstruction. In most patients with a history of radiotherapy, autologous reconstruction is required because the complication rate with implant-based reconstruction is greater than 40% (Barry & Kell 2011; Kronowitz & Robb 2009). The advantages and disadvantages of the different techniques enter into the patient’s final decision as concerns delayed reconstruction (Alderman et al. 2002; Cordeiro & McCarty 2006; Rouzier et al. 2000).

For the majority of patients, the decision not to have nonreconstruction was reportedly a personal choice. The major reason was refusal of further surgery. Clearly it is legitimate for patients to be reluctant to have surgery that is not necessarily perceived to be an integral part of treatment of the disease as such. Patients’ concerns about the number of surgeries and the duration of recovery may outweigh the potential benefits in terms of cosmesis and quality of life.

Acceptance of body asymmetry by the patient herself and/or by her partner was frequently reported as the reason for not having reconstruction. This finding points to a potential weakness in the study, i.e. the fact that the questionnaire was sent out several years after the initial decision concerning reconstruction was taken. As a result, the responses probably reflect to some extent what patients considered to be the reason(s) for not having reconstruction at the time of filling out the questionnaire. Nevertheless, the decision is not necessarily taken ‘once and for all’. The availability of delayed reconstruction means that the option may remain open for several years after mastectomy, as illustrated by the fact that, at the time of analysis in our study, the median time to delayed reconstruction was about 1½ years, with a maximum of almost 6½ years. For this reason, while we recognize it as a potential weakness, we do not believe that it undermines the validity of the finding.

The risk of complications was reported as the reason for not having reconstruction by almost 30% of patients, which is lower than the percentages seen in other studies (Shameem et al. 2008; Alderman & Jagsi 2010). Endorsement of this reason indicates that the patients were aware of the potential complications. Although the source of the information could not be ascertained, a recent review by Potter et al. highlights problems in reporting outcomes in reconstruction, particularly as concerns the assessment of surgical complications (Potter et al. 2010).

Fear of masking recurrence of the disease was reported by 18% of patients. Considerably higher percentages have been reported elsewhere (Shameem et al. 2008; Alderman & Jagsi 2010), even though studies going back as far as the mid-1990s do not support the fear that reconstruction might interfere with postoperative cancer surveillance. This finding has obvious implications in terms of patient counseling, suggesting that, with better information, patients might overcome their fear.

Age was not listed among the multiple-choice responses on the questionnaire, but was added in the free-text section ‘Other reason(s)’ by almost a quarter of patients, making this an important finding. The reasons why age in itself should be a major factor have not been fully elucidated, but certainly merit further investigation.

As expected, financial considerations were not a major factor since, under the publicly funded healthcare system in France, patients do not have to pay for the procedure, theoretically at least. In practice, however, access to reconstruction in public institutions is restricted by the limited number of surgeons and availability of operating theaters. As a result, a number of patients in France are led to consult in private hospitals, which entails additional costs, borne by the patient herself or by patient-funded complementary health insurance. Financial cost as a reason for not having reconstruction, as reported by 14% of patients, reflects a problem of access to the procedure within our institution.

Finally, post-mastectomy pain was reported by 6.5% of patients. Similar to acceptance of body asymmetry, this reason for not having reconstruction reflects the ongoing nature of the decision and is relevant only to patients who were offered or were considering delayed reconstruction. It suggests the need for better control of complications following mastectomy.

Sixty-one patients completed the questions related to the source of and satisfaction with information on reconstruction. While the questionnaire did not preclude patients from citing more than one response, only one respondent cited two sources of information, pointing to another potential weakness in our findings. More than three-quarters of patients reported that the information had been received from a medical source, most often (63%) a source within Institut Curie: a surgeon, a plastic surgeon, a doctor or nurse, or from the Institut website or review (Table 6), however the actual proportion is almost certainly even higher. Although provision of information is not recorded in patients’ files, standard procedures at Institut Curie make it nearly impossible for a patient who is eligible for reconstruction not to be informed of this option.

It is therefore noteworthy that, in 40% of patients overall, the decision was taken after receiving information they considered to be ‘entirely unsatisfactory’. A similar figure was found in the National Mastectomy and Breast Reconstruction Audit in 2010, in which 41.6% of mastectomy-only patients reported they had been “given no information” (Mayor 2010). As concerns our finding, it is probably not only a question of quantity or even of quality, but also of timing and the method of delivering information prior to mastectomy, i.e. at a time when many patients may not be highly receptive.

Another study has been set up to investigate the provision of information in our institution, involving patients and care-givers on an equal footing.

## Conclusions

The majority of patients with breast cancer who undergo mastectomy do not proceed with breast reconstruction. The decision involves multiple factors, some biological and socio-demographic, as well as personal choice. Almost two-thirds of patients were unsatisfied with the information they received. We believe efforts must be made to improve the type and timing of delivering information in order to ensure that patients receive the information they require. By better understanding the factors that the patient takes into consideration in making her decision, we can adapt the information to serve the patient’s personal needs and to support the patient in making an informed personal choice on approaches to reconstruction following mastectomy. Future studies must be directed with patients themselves to improve the type and timing of delivering information on different ways of reconstruction.

### Previous presentations of this manuscript

Presented in part at the 33ème Journées de la Société Française de Sénologie et Pathologie Mammaire, November 8–13, 2011, Marseille, France.

Presented in part at the 8th European Breast Cancer Conference, March 21–24, 2012, Vienna, Austria.

### Ethical approval

This study was conducted according to the institutional and ethical rules concerning research on tissue specimens and patients and exempted from requiring informed consent from the patients.
